# Prevention of Incisional Hernias after Open Abdomen Treatment

**DOI:** 10.3389/fsurg.2018.00011

**Published:** 2018-02-26

**Authors:** Frederik Berrevoet

**Affiliations:** ^1^Ghent University Hospital, Department of General and HPB Surgery and Liver Transplantation, Ghent, Belgium

**Keywords:** open abdomen, prevention, negative pressure therapy, botulinum toxin, component separation

## Abstract

Management of a patient with an open abdomen is difficult, and the primary closure of the fascial edges is essential to obtain the best patient outcome, regardless of the initial etiology of the open abdomen. The use of temporary abdominal closure devices is nowadays the gold standard to have the highest closure rates with mesh-mediated fascial traction as the proposed standard of care. However, the incidence of incisional hernias, although much more controlled than when leaving an abdomen open, is high and reaches up to 65%. As shown for other high-risk patient subgroups, such as obese patients, patients with an abdominal aneurysm, and patients with former -ostomy sites, the prevention of incisional hernias might be key to further optimize patient outcomes after open abdomen treatment. In this overview, current available modalities to decrease the incidence of incisional hernia are discussed. Most of these preventive options have been shown effective in giant ventral hernia repair and might work effectively in this patient cohort with open abdomen as well.

## Introduction

Open abdomen therapy is sometimes necessary to save lives in trauma and nontrauma surgical indications (1). Different reasons might be the underlying cause for the choice of the surgeon to leave the abdominal cavity open: visceral edema and inability to close the fascial edges, fear for intraabdominal hypertension and abdominal compartment syndrome, a planned relaparotomy or sepsis with the need for intraabdominal negative pressure therapy.

Management of the open abdomen in the postoperative phase is difficult, because of fluid shifts, loss of domain, and the underlying metabolic derangements. Complications associated with the open abdomen technique also have been reported, with the development of entero-atmospheric fistulas and infectious complications in this severely ill patient population. One can postulate that patients who require open abdomen management are at a substantial risk for systemic infectious complications, because of the use of temporary abdominal closure (TAC) techniques, a prolonged inflammatory phase, and the acquired immunocompromised state. These extraabdominal secondary infectious complications may play a considerable role in whether primary closure is achieved (2). The high burden of comorbidities and the severe illness of these patients may also lead to the prolongation of open abdomen therapy and more difficulties to finally close the abdominal cavity.

Nowadays, different TAC techniques are frequently used, and a real consensus on which technique to be used has not yet been reached (3, 4). The TAC dressing should ideally cover the intraabdominal contents to maintain a physiological environment in an active way, prevent evisceration, prevent adhesions, protect the bowel wall from injury, remove the excess of wound fluid, bacteria, and cytokines in an active way, and should also facilitate subsequent abdominal wall closure as early as possible, preferably within 2 weeks.

The “old-classic” Bogota bag, a silastic or a silicon sheet, just a skin approximation or a zipper-type of closure, might be valuable in cases with damage control surgery and intraabdominal bleeding, in which definitive closure is anticipated within the next 2 or 3 days. No fluid removal is possible, and as there is no fascial traction present, these devices should not be left in place for more than 3–4 days.

A Wittmann Patch, dynamic retention sutures, or inlay mesh placement are examples of a more dynamic closure technique, which can be used as a temporary TAC system to prevent lateral fascial retraction and permit adjustments according to the intraabdominal pressure. They facilitate reoperation, but do not allow for the effective drainage of intraabdominal fluids and bacteria. Furthermore, they might be related to an increased rate of enterocutaneous fistula (5). In addition, there is a concern that the sutures placed on the fascia might cause ischemic damage to the edges, making the definitive closure more difficult.

In case of an infected or contaminated abdominal cavity, intraabdominal negative pressure treatment plays an important role: it is able to remove peritoneal fluid and inflammatory mediators, reduces the concentration of cytokines in the bloodstream, and partially facilitates the approximation of the fascial edges. Among the different NPT techniques (Barker vacuum pack, VAC abdominal dressing system™, and ABThera™), the ABThera dressing system seems to achieve better results in terms of survival, enterocutaneous fistula rate, and peritoneal fluid removal (6). However, attention has to be paid to incomplete hemostasis and high-risk gastrointestinal anastomoses. Dressing chances are performed every 1–3 days or even more frequently in case there is an abdominal contamination or an infection. Moreover, the combination of both negative pressure therapy and fascial traction has been demonstrated to be ideal in managing patients who may not achieve primary facial closure with intraabdominal NPT alone (7, 8). A definitive closure can then be obtained only when the abdominal acute condition and bowel edema are resolved and the intraabdominal pressure remains under 25–30 mmHg (3).

## Open Abdomen Closure

In case patients have been treated adequately for an open abdomen, the final closure of the abdomen should be achieved within 10–14 days after the initial surgery. However, the evidence on how to perform an optimal fascial closure in these patients is scarcely documented. Only three observational cohort studies described the fascial closure method for both septic and non-septic patient conditions, but no data were available on, e.g., burst abdomen or fistula formation (9–11). In these studies, both continuous and interrupted suture techniques were used, but there is a high heterogeneity among these studies as it concerns case series only. Sörelius et al. reported on 30 patients treated with OA among a cohort of 1,041 patients treated for an aortic aneurysm (9). Four died before abdominal closure, while primary delayed fascial closure was achieved in all survivors. Incisional hernias occurred in 9 of 15 patients (60%); only three were symptomatic. They used mesh-mediated fascial traction, and at the time of final closure, the entire mesh was removed and the fascia was closed with a running 0 polydioxanone suture, by means of a standardized suturing technique aiming at a suture length to wound length ratio of at least 4:1. By contrast, Brandl et al. published their cohort of 209 OA patients in which the final fascial closure technique was determined by the surgeon in charge and recorded as continuous or interrupted, using absorbable or nonabsorbable suture materials (10). In case of continuous suturing, they followed the 4:1 ratio rule. In a few patients (7.3%) in which direct fascial closure was not possible, they used a biological or a synthetic mesh to bridge the defect. In their analysis, they showed a trend toward a higher incisional hernia rate for the interrupted rapidly absorbable sutures (41 versus 10% for rapidly absorbable and slowly resorbable sutures, respectively). In another cohort of 87 patients reported by Fortelny et al., dynamic fascial sutures were combined with negative pressure therapy; closure was achieved in 78% of patients after 12.6 days and 4.3 reoperations (11). Fifty-five patients (63.2%) were closed using a non-resorbable or slowly resorbable running suture, while eight patients (9.2%) had an interrupted suturing technique. No further details on the outcomes regarding the different suturing technique and materials were reported in this study.

Regarding fascial closure, also other reconstructive techniques have been described, using different flap reconstructions as the bipedicled myofascial oblique rectus abdominis flap and the bilateral anterior rectus abdominis sheath turnover flap. However, besides the surgical technique, very limited long-term data have been reported regarding both complication and incisional hernia rates (12, 13).

Therefore, according to the limited data available and no formal consensus in the literature, a continuous fascial closure seems most logical, in accordance with the guidelines of the European Hernia Society on abdominal wall closure, using slowly resorbable sutures in a suture to wound length ratio of 4:1 as initially described by Israellson and coworkers (14, 15).

## Incidence of Incisional Hernia after Open Abdomen Treatment

Despite all efforts to finally obtain full fascial closure in OA patients, the long-term follow-up of these patients in terms of incisional hernia rate is scarce, but rather worrisome. Again, only three studies reported on long-term outcome data, two of which were prospective (16–18). The total number of patients is low (range 14–50), and the incidence of incisional hernias ranged from 21% at 21 months to 54% after 5 years of follow-up. The repair rate differed and was 33 and 42% in the prospective cohorts, respectively.

Bjarnason and coworkers reported their 1-year follow-up after a mesh-mediated fascial closure in combination with NPT and described 66% of incisional hernias in these patients using CT evaluation (19). Remarkably, four patients who had early definitive fascial closure with permanent mesh reinforcement did not show an incisional hernia. Suturing of the mesh to the fascial edges as in the mesh-mediated fascia traction method may lead to incisional hernia development, due to possible greater traction on the fascial edges. Therefore, despite the fact that more patients can be closed after OA treatment and TAC therapy using fascial traction in combination with NPT, the focus for these patients should now more and more be on how to prevent incisional hernias from developing after final fascial closure in this patient population.

## Prevention of Incisional Hernia Development after OA Management

In general, the prevention of incisional hernia can be achieved using a state-of-the-art suturing technique with an SL:WL ratio of 4:1 or more, using small bites instead of large ones on both fascial edges, limiting traction on the fascial edges at the time of the final fascial closure and by using a mesh at the time of closure. If this is true for different patient settings as patients with parastomal hernias, midline laparotomy closure in high-risk patients with previous ostomies, AAA patients, and obese patients, the same could be true for the high-risk patient population that OA patients resemble. However, as OA patients are mostly severely ill, malnourished, and on top of that, the fascial edges are generally more fragile due to previous OA treatment, the usage of a correct surgical technique with adequate length ratio, and small bites might not be sufficient in these patients, as is shown by the high rates of incisional formation in these patients. Therefore, other available surgical techniques in abdominal wall reconstruction might be appropriate to apply in OA fascial closure. The main objective should probably be the release of tension on the fascial edges to allow for a “tension-free” closure of the fascia. Different options will be discussed here, although evidence on these techniques for this specific indication is almost nonexisting.

### Component Separation Techniques (CSTs)

Component separation technique is nowadays a well-known surgical technique, with multiple anatomical variants (20, 21). It will allow for the medial advancement of the fascia edges up to 10 cm on each side around the umbilicus, so that defects up to 15–20 cm in width can be closed with an augmentation technique. Therefore, in OA patients, this technique can be considered as a useful adjunct to achieve the final fascial closure. Two observational studies using CST and two observational studies using other fascio-myoplasty techniques could be retrieved from the literature (12, 22, 23). However, these reports document only on these techniques with an indication to close the fascial edges at the time of final closure and not in relation to prophylaxis. For the flap techniques, only Kushimoto reported on long-term data in these patients with half of the patients showing a mid-abdominal bulging but no incisional hernias in 10 patients after a 65-month follow-up without further specification (12). The same is true for the papers reporting on CST for OA patients. Rasilainen et al. published on 16 patients of which 9 patients were treated using CST at the time of final fascial closure and 7 during ongoing open abdomen management (23). All patients with CST at the time of final closure could be primarily closed. Out of eight patients who survived and had CST at the time of fascial closure, 25% developed an incisional hernia. Sriussadapron et al. also reported on eight patients who had closure of “acute abdominal wall defects” using CST, but using various modifications of the CST, making comparison difficult (22). No report on incisional hernia development in these patients was done. Despite these very low numbers, a recent review by Sharrock et al. stated that the use of CST may be a valid alternative to delayed primary closure following damage control laparotomy in trauma patients (24). Regarding the combination of CST and mesh augmentation, there is no evidence supporting this; however, when considering the results of CST only in large incisional hernia repair, which are rather poor (25), a combination of CST and mesh augmentation might be an option.

In the study by Bjarnason et al., eight patients had early fascial closure with synthetic mesh reinforcement, of which four survivors showed no incisional hernia later on, but again, this synthetic mesh was only used to get the abdominal wall closed and not as “additional prophylaxis” (19). Based on this evidence, it is still very much unclear whether CST should be advocated to be used preventively, in addition to primary fascial closure in order to release tension and decrease incisional hernia development. Furthermore, the use of CST in these patients will burn a possible “bridge” for later complex hernia repair in case an incisional hernia will develop. It is clear that more data and well-designed studies are needed to clarify the role of CST as a preventive tool in OA patients.

### Use of Botulinum Toxin Type A (BTA)

As proven methods for decreasing the rate of planned ventral hernia provide tension in the midline to counter the effects of lateral abdominal wall muscular retraction (26–28), BTA is an FDA-approved neuromodulating agent that has now become increasingly popular in abdominal wall reconstruction (29, 30). This toxin functions by blocking the release of acetylcholine and pain modulators and is also referred to as a “chemical components separation” technique, which takes advantage of the flaccid paralysis created after BTX injection. If this flaccid paralysis can provide decreased midline abdominal wall tension, then, theoretically, the rate of primary fascial closure will increase, or as meant in prevention, the number of incisional hernias after OA treatment might decrease.

The only report involving BTX in open abdomen management has been published by Zielinski et al. (31). They recommend the use of either a negative pressure dressing or a Wittmann Patch as a TAC device at the time of the initial laparotomy, to ensure midline tension. After stabilization of the patient, at the time of reexploration or changing the NPT dressing, the patient can be prepared to allow for sufficient space for ultrasound-guided BTX injection. Three-hundred units of BTX are reconstituted in 150 cc of injectable normal saline and injected according to the protocol as described by Zendejas et al. (32). They reported on 18 patients for who the primary fascial closure rate was 83%. Nine patients underwent chemical components separation within 24 h of their initial OA procedure. Of these, 89% (8/9) achieved primary fascial closure, without complications related to BTX. The presence of an open abdomen allows for biomechanical forces to develop that ordinarily are counteracted from the normal anatomical structure of the abdominal wall. The lateral abdominal wall musculature (external oblique, internal oblique, and transversus abdominus) creates a lateral retraction force that is opposed centrally at the linea alba. The success of negative pressure dressings and the Wittmann Patch in enhancing the ability to perform primary fascial closure is likely related to the opposition of this lateral wall abdominal retraction. Logically, therefore, if a safe method of decreasing the lateral abdominal wall retraction can be developed, the ability to perform primary fascial closure could improve. But again, no data are available on using BTX as a preventive measure, for example, at the time of final primary closure to explore the benefit of this decreased tension at the level of the linea alba in terms of incisional hernia development.

### Mesh

Regarding the closure of an open abdomen, both the synthetic mesh-mediated fascial traction in combination with NPT has been advocated, with currently the best outcomes reported, as well as the use of (resorbable) meshes as an adjunct to primary fascial closure during OA management. Four observational studies (33–36) reported on using mesh at the time of primary fascial closure. Regarding the presence of an incisional hernia, all together, 98 patients were studied with an incidence of incisional hernias of around 20%. It is clear that the surgical site occurrences and infection rate are increased when using mesh (up to 30%). Therefore, there is very limited evidence that supports the use of mesh reinforcement after fascial closure in an OA. On the other hand, in comparison, again, with other high-risk population for the development of incisional hernias, it seems logical to extrapolate these data to the OA population in order to decrease the percentage of incisional hernias. There is absolutely no data to propose a certain position of the mesh, i.e., onlay, sublay, or intraperitoneal mesh positioning. However, looking at the technical difficulty, the risk for mesh infection, and comparing different other indications for prophylactic mesh use, the use of a mesh in an onlay position seems appropriate (37). As it is also not clear up to now, whether we might need definitive or temporary mesh reinforcement after primary fascial closure in these patients, the type of mesh, synthetic, biological, or biosynthetic (or a hybrid or a long-term resorbable synthetic), is not known and cannot be recommended at this point of time. When considering the higher percentage of SSO and SSI when using mesh, this might be a reason for surgeons to choose for a biological or long-term resorbable synthetic mesh (TIGR™, Bio-A™, or Phasix™). On the other hand, as the incidences of SSO reported in the trials for parastomal mesh prevention are so low (38), a permanent synthetic mesh might work well and might give the best results on the long term, regarding incisional hernia rates.

## Conclusion

Considering the OA patient population, abdominal wall closure is our main priority, and the use of TAC techniques seems advocated to using a combination of (dynamic) fascial traction and NPT. However, as the rate of incisional hernia formation is extremely high among survivors in this group of patients, prevention at the time of fascial closure seems mandatory as well as logical. As the proposed surgical techniques for closure of the abdominal wall, as proposed by the EHS, do not really apply to this subset of patients with an open abdomen, mesh reinforcement, CSTs, and the use of BTA might have their place in prevention. However, as the literature on this specific issue is lacking, only data extrapolation from other high-risk groups seems possible, with all its inherent limitations. No definitive conclusions can be made regarding their use in terms of prevention of incisional hernia development at the time of primary fascial closure in open abdomen patients. More future studies, together with register data, have to tackle this issue and could help us to further refine open abdomen management.

## Author Contributions

The author confirms being the sole contributor of this work and approved it for publication.

## Conflict of Interest Statement

The author declares that the submitted work was carried out in the absence of any personal, professional, or financial relationships that could potentially be construed as a conflict of interest.
